# Ischemic Stroke in Critically Ill Patients with Malignancy

**DOI:** 10.1371/journal.pone.0146836

**Published:** 2016-01-11

**Authors:** Jeong-Am Ryu, Oh Young Bang, Gee Young Suh, Jeong Hoon Yang, Daesang Lee, Jinkyeong Park, Joongbum Cho, Chi Ryang Chung, Chi-Min Park, Kyeongman Jeon

**Affiliations:** 1 Department of Critical Care Medicine, Samsung Medical Center, Sungkyunkwan University School of Medicine, Seoul, Republic of Korea; 2 Department of Neurology, Samsung Medical Center, Sungkyunkwan University School of Medicine, Seoul, Republic of Korea; 3 Division of Pulmonary and Critical Care Medicine, Department of Medicine, Samsung Medical Center, Sungkyunkwan University School of Medicine, Seoul, Korea; 4 Division of Cardiology, Department of Medicine, Samsung Medical Center, Sungkyunkwan University School of Medicine, Seoul, Republic of Korea; 5 Department of Surgery, Samsung Medical Center, Sungkyunkwan University School of Medicine, Seoul, Republic of Korea; University of Ulm, GERMANY

## Abstract

**Background:**

Cerebrovascular diseases are a frequent cause of neurological symptoms in patients with cancer. The clinical characteristics of ischemic stroke (IS) in patients with cancer have been reported in several studies; however, limited data are available regarding critically ill patients with cancer who develop IS during their stay in the intensive care unit (ICU).

**Methods:**

All consecutive patients who underwent brain magnetic resonance imaging (MRI) for suspicion of IS with acute abnormal neurologic symptoms or who developed signs of IS while in the ICU were retrospectively evaluated. We compared the clinical characteristics and diffusion-weighted imaging (DWI) lesion patterns between patients finally diagnosed as having or not having IS.

**Results:**

Over the study period, a total of 88 patients underwent brain MRI for suspicion of IS, with altered mental status in 55 (63%), hemiparesis in 28 (32%), and seizure in 20 (23%). A total of 43 (49%) patients were ultimately diagnosed with IS. Multiple DWI lesions (41%) were more common than single lesions (8%). The etiologies of IS were not determined in the majority of patients (n = 27, 63%). In the remaining 16 (37%) patients, the most common aetiology of IS was cardioembolism (n = 8), followed by large-vessel atherosclerosis (n = 3) and small-vessel occlusion (n = 2). However, brain metastases were newly diagnosed in only 7 (8%) patients. Univariate comparison of the baseline characteristics between patients with or without IS did not reveal any significant differences in sex, malignancy type, recent chemotherapy, vascular risk factors, or serum D-dimer levels at the time of suspicion of IS. Thrombotic events were more common in the IS group than in the non-IS group (*P* = 0.028). However, patients who were ultimately diagnosed with IS had more hemiparesis symptoms at the time of suspicion of IS (*P* = 0.001). This association was significant even after adjusting for potentially confounding factors (adjusted odds ratio 5.339; 95% confidence interval, 1.521–19.163).

**Conclusions:**

IS developed during ICU stays in critically ill patients with cancer have particular features that may be associated with cancer-related mechanism.

## Introduction

Critically ill patients admitted to intensive care units (ICUs) are at risk for neurologic complications [1]. These complications occur as the result of critical illness, intensive care therapies and procedures, and/or medical or surgical conditions [2]. Previous studies have shown that 12~33% of all patients admitted for non-neurologic reasons experience neurologic complications that are detrimental to their outcomes [3,4]. Although some neurologic conditions such as cognitive dysfunction [5] and neuromuscular complications [6] are frequently studied in patients in intensive care, other complications such as cerebrovascular disease have not been well studied.

Cerebrovascular disease occurs often in patients with cancer [7]. An autopsy-based study found that approximately 15% of all patients with cancer experienced thromboembolic events, including ischemic stroke (IS), during their clinical course [8]. Systemic cancer is related to IS via various mechanisms [9]. Specifically, cancer-related mechanisms such as coagulopathy and tumour occlusion are a common cause of IS in patients with cancer [10–14]. In addition, treatment modalities such as chemotherapy, radiotherapy, and hormone therapy may also increase the risk of IS [13–15]. However, little is known regarding the causes and outcomes of IS in critically ill patients with cancer.

Therefore, we conducted a retrospective observational study with critically ill patients with cancer who were admitted to a medical ICU. Our goal was to evaluate the clinical characteristics of IS that developed during their ICU stays.

## Methods

This retrospective study was performed with a cohort of critically ill patients with cancer who underwent brain magnetic resonance imaging (MRI) for suspicion of IS during their ICU stay. Patients were treated in the oncology medical ICU of Samsung Comprehensive Cancer Center of Samsung Medical Center (a 1961-bed, university-affiliated, tertiary referral hospital in Seoul, South Korea) from March 2010 to February 2014. This study was approved by the Institutional Review Board of Samsung Medical Center and full permission was given to review and publish information from patient records. The requirement for informed consent was waived due to the retrospective nature of the study.

All consecutive critically ill patients with cancer admitted to the oncology medical ICU were considered for this study if they underwent brain MRI for suspicion of IS with acute neurologic symptoms or exhibited signs of IS during their ICU stay. Patients were excluded if they had a history of trauma, surgery, or a chronic neurologic deficit on ICU admission.

The diagnosis of IS was made by reviewing brain MRI scans. A positive diagnosis was made when the ischemic regions had decreased apparent diffusion coefficients (ADCs) and high signal intensities on diffusion-weighted imaging (DWI) [16]. The DWI patterns were classified as single lesions or multiple lesions. Multiple lesions were subdivided into the following categories: single arterial territory, small lesions involving multiple arterial territories, and small and large disseminated lesions. The IS subtype was classified according to the Trial of Org 10172 in Acute Stroke Treatment (TOAST) system [17]. Briefly, stroke etiologies were divided into the following 5 categories: (1) large vessel atherosclerosis: significant stenosis (>50%) or occlusion of an appropriate major brain artery or branch cortical artery due to atherosclerosis; (2) cardioembolic: an embolus arising in the heart or related risk factors such as atrial fibrillation, prosthetic cardiac valve, severe left ventricular dysfunction, and acute myocardial infarction (<3 weeks); (3) small-vessel occlusion: a relevant brainstem or subcortical hemispheric infarction < 1.5 cm in size on neuroimages; (4) other, including coagulopathy or perioperative stroke; and (5) undetermined.

### Data Collection

The following data were extracted from electronic medical records on ICU admission: age, sex, vascular risk factors for stroke, malignancy status (including malignancy type), disease status and extensiveness, and reason for ICU admission. Since this study included only patients with malignancies, previous definitions associated with cancer status were used [18–21]. Patients experiencing a relapse in their malignancy following intensive front-line chemotherapy or who failed to respond to initial chemotherapy were considered to be in a relapsed/refractory state [20,21]. The extensiveness of the malignancy was classified according to the tumour extent and involvement of major organs, as reported previously [18–21]. Extensive disease was defined as stage III or IV for lymphoma and as metastatic or locally extensive disease for solid malignancies. For haematological malignancies, extensive disease was defined as >80% blasts in bone marrow, >25,000 blasts/μL in peripheral blood, or the need for leukapheresis [20,21]. Major organ involvement was defined as a pathologically confirmed or radiologically suspected invasion of the brain, heart, lung, liver, or kidney [19]. Illness severity was scored using the Simplified Acute Physiology Score 3 (SAPS 3) and Sequential Organ Failure Assessment (SOFA) systems.

At the time of brain MRI, the following data were extracted from medical records: blood pressure as recorded within the last 24 hours, laboratory data, neurologic symptoms or signs, and other clinical data. Previous or concomitant thrombotic events, including ischemic stroke, myocardial infarction, deep vein thrombosis, and pulmonary embolism were also evaluated. Disseminated intravascular coagulation (DIC) was identified using the International Society on Thrombosis and Hemostasis (ISTH) DIC scoring system [22].

### Statistical Analyses

All data are presented as medians and interquartile ranges (IQRs) for continuous variables and as numbers (percentages) for categorical variables. Data were compared using the Mann-Whitney U test for continuous variables and the chi-square test or Fisher’s exact test for categorical variables. Multiple logistic regression analysis was used to identify independent predictors of IS in critically ill patients with cancer. In this analysis, the estimated odds ratio (OR) and 95% confidence interval (CI) for each parameter were calculated. Variables with a *P* value less than 0.2 by univariate analysis and all a priori clinically relevant variables were entered into the forward stepwise multiple logistic regression model. All tests were 2 sided, and *P* values < 0.05 were considered to indicate statistical significance. Data were analysed using IBM SPSS statistics 20 (IBM, Armonk, NY).

## Results

Over the study period, a total of 2,258 critically ill patients with cancer were admitted to the oncology medical ICU. Of these, 88 patients with cancer who underwent brain MRI for suspicion of IS with acute neurologic symptoms or signs during their ICU stay were included in the final analysis.

The patient baseline characteristics are presented in Table 1. Of the 88 patients, 51 were male (58%); the median age was 63 (range 53–69) years. A total of 55 (63%) patients suffered from hematologic malignancies, including leukaemia (n = 18), lymphoma (n = 22), multiple myeloma (n = 10), and myelodysplastic syndrome (n = 3). The remaining 33 (38%) patients had solid tumours, including lung cancer (n = 18), hepatic cancer (n = 3), gastric cancer (n = 3), and brain cancer (n = 2). Respiratory failure (42%) and severe sepsis or septic shock (39%) were the most common causes of ICU admission. Hypertension (40%) and smoking (35%) were the most common vascular risk factors for IS.

**Table 1:** Baseline characteristics of 88 critically ill cancer patients who underwent brain magnetic resonance imaging for suspicion of ischemic stroke during their stay in the intensive care unit.

Characteristics	No. of patients (%) or median (IQR)
Age, years	63 (53–69)
Gender, male	51 (58)
Type of malignancy	
Solid tumour	33 (38)
Hematologic malignancy	55 (63)
Status of malignancy on ICU admission	
First presentation	33 (38)
Relapsed/refractory	45 (51)
Extensive disease	38 (43)
Major organ involvement	21 (24)
Stem cell transplantation	13 (15)
Duration of malignancy, months	3.7 (0.8–11.7)
Major reasons for ICU admission	
Respiratory failure	37 (42)
Severe sepsis or septic shock	34 (39)
Cardiovascular	5 (6)
Neurological	6 (7)
Other	6 (7)
Clinical status on ICU admission	
Recent chemotherapy prior to ICU admission within 4 weeks	44 (50)
Need for mechanical ventilation	58 (66)
Need for vasopressor support	33 (38)
Need for renal replacement therapy	7 (8)
D-dimer levels (μg/mL)	4.51 (2.18–10.59)
Vascular risk factor	
Hypertension	35 (40)
Diabetes mellitus	20 (23)
Ex and current smoking	31 (35)
Ischemic heart disease	3 (3)
Hypercholesterolemia	5 (6)
Atrial fibrillation	19 (22)
Alcohol abuse	4 (5)
Family history of stroke	7 (8)
Previous thrombotic event	10 (11)
Ischemic stroke	3 (3)
Myocardial infarction	4 (5)
Deep vein thrombosis	2 (2)
Pulmonary thromboembolism	1 (1)
Severity of illness	
SAPS3	65 (53–78)
SOFA	9 (7–12)

IQR, interquartile range; ICU, intensive care unit; SAPS, simplified acute physiology score; SOFA, sequential organ failure assessment

The clinical characteristics of the patients at the time of brain MRI for suspicion of IS are presented in Table 2. The most common symptom or sign for clinical suspicion of IS was altered mental status, which was present in 55 (63%) patients. This symptom was followed by hemiparesis in 28 (32%), seizure in 20 (23%), abnormal movement in 6 (7%), and anisocoric pupils or an abnormal pupil reflex in 3 (3%) patients. Overlap of symptoms and signs was observed in 33% of the patients.

**Table 2:** Clinical characteristics of 88 critically ill cancer patients at the time of brain magnetic resonance imaging for clinical suspicion of ischemic stroke in the intensive care unit.

Characteristics	No. of patients (%) or median (IQR)
Neurologic symptoms or signs	
Decreased mentality or delirium	55 (63)
Hemiparesis	28 (32)
Seizure	20 (23)
Abnormal movement	6 (7)
Anisocoric pupil or abnormal pupil reflex	3 (3)
Abnormal respiratory pattern	2 (2)
Other	7 (8)
Time interval from ICU admission to brain MRI, days	4.4 (1.1–12.4)
Recent chemotherapy	44 (50)
Existing central vein catheter	71 (81)
Fungal infection	21 (24)
Infective endocarditis	3 (3)
Anticoagulation use	20 (23)
Antiplatelet use	3 (3)
D-dimer levels (μg/mL)	3.76 (1.73–10.19)
Disseminated intravascular coagulation	3 (3)

IQR, interquartile range; ICU, intensive care unit; MRI, magnetic resonance imaging

Of the 88 patients who underwent brain MRI for suspicion of IS, 43 (49%) had a final diagnosis of IS. The brain MRI findings of these patients are summarised in Table 3. Multiple lesions were more common (41%) than single lesions (8%). The etiologies of stroke were identified in 16 (37%) patients, including cardioembolism (n = 8), large-vessel atherosclerosis (n = 3), small-vessel occlusion (n = 2), and other (n = 3). However, the stroke etiologies in the remaining 27 (63%) patients were not determined. In addition, brain metastases were newly diagnosed in 7 (8%) patients.

**Table 3:** Brain magnetic resonance imaging findings in 43 patients diagnosed with ischemic stroke and 45 non-ischemic stroke patients during their stay in the intensive care unit.

Brain MRI findings	No. of patients (%)
Ischemic stroke patients	
Single lesion	7 (8)
Multiple lesions	36 (41)
A single arterial territory	1 (1)
Small lesions involving multiple arterial territories	18 (20)
Small and large disseminated lesions	17 (19)
Non-ischemic stroke patients	
Pathologic brain MRI findings	32 (36)
Brain metastasis*	7 (8)
Old stroke lesion	7 (8)
Posterior reversible encephalopathy syndrome	4 (5)
Intracranial haemorrhage (1 gyral SAH, 2 SDH)	3 (3)
Seizure-related change	3 (3)
Primary brain tumor	3 (3)
Other	5 (6)
Normal brain MRI findings	13 (15)

MRI, magnetic resonance imaging; SAH, subarachnoidal haemorrhage; SDH, subdural haemorrhage

*Newly diagnosed brain metastasis

Univariate comparisons are presented in Table 4. Specifically, patient baseline characteristics at the time of suspicion of IS were compared. In addition, the outcomes of patients with IS vs those of patients without IS were compared. No significant differences were observed regarding sex, malignancy type, recent chemotherapy, vascular risk factors, or serum D-dimer level at the time of suspicion of IS. Thrombotic events were more common in the IS group than in the non-IS group (*P* = 0.028). However, patients finally diagnosed with IS had more hemiparesis symptoms at the time of suspicion of IS (*P* = 0.001). The non-IS group had more seizures (*P* = 0.001). After adjusting for potentially confounding factors, hemiparesis (adjusted OR 5.339; 95% CI, 1.521–19.163) was found to be independently associated with IS in patients who underwent brain MRI for suspicion of IS in the oncology medical ICU. In contrast, seizure was inversely associated with IS (adjusted OR 0.141; 95% CI 0.027–0.736). No significant differences were observed regarding the length of ICU stay (*P* = 0.299), ICU mortality (*P* = 0.114), or in-hospital mortality (*P* = 0.085) between patients with IS vs patients without IS.

**Table 4:** Comparisons of clinical characteristics at the time of brain magnetic resonance imaging for clinical suspicion of ischemic stroke (IS) and outcomes between patients finally diagnosed as having or not having IS.

	IS (n = 43)	Non-IS (n = 45)	*P* value
Age, years	64.5 (56–69)	62.5 (40–70)	0.110
Gender, male	26 (61)	25 (56)	0.641
Type of malignancy			0.956
Solid	16 (37)	17 (39)	
Hematologic	27 (63)	28 (62)	
Resent chemotherapy	22 (51)	22 (49)	0.831
Vascular risk factor			
Hypertension	17 (40)	18 (40)	0.964
Ex and current smoking	14 (33)	17 (38)	0.608
Ischemic heart disease	2 (5)	1 (2)	0.530
Hypercholesterolemia	2 (5)	3 (7)	0.683
Atrial fibrillation	8 (19)	11 (24)	0.506
Diabetes mellitus	11 (26)	9 (20)	0.532
Alcohol abuse	3 (7)	1 (2)	0.355
Thrombotic event	10 (23)	3 (7)	0.028
Previous thrombotic event^1^	7 (16)	3(7)	0.191
Concomitant pulmonary thromboembolism	2 (5)	0 (0)	0.236
Concomitant deep vein thrombosis	5 (12)	1 (2)	0.106
Neurologic symptoms or signs			
Acute change in mental status	28 (65)	27 (60)	0.620
Seizure	3 (7)	17 (38)	0.001
Hemiparesis	21 (49)	7 (16)	0.001
Abnormal movement	4 (9)	2 (4)	0.429
Anisocoric pupil or abnormal pupil reflex	1 (2)	2 (4)	0.584
Other	2 (5)	4 (9)	0.677
Antiplatelet use	2 (5)	1 (2)	0.612
Anticoagulant use	10 (23)	10 (22)	0.908
D-dimer levels (μg/mL)	5.66 (2.13–11.80)	3.53 (2.17–8.53)	0.319
Disseminated intravascular coagulation	3 (7)	0 (0)	0.112
Outcomes			
ICU mortality	19 (45)	13 (29)	0.114
In-hospital mortality	34 (81)	29 (64)	0.085
Length of stay in ICU, days	15.2 (9.2–19.5)	12.2 (6.0–20.1)	0.299

Data are expressed as medians (interquartile range) or frequencies (%). IQR, interquartile range; ICU, intensive care unit.

^1^Previous thrombotic events include ischemic stroke, myocardial infarction, deep vein thrombosis and pulmonary embolism.

## Discussion

Here we evaluated the clinical characteristics of IS in critically ill patients with cancer. In this retrospective observational study, approximately half of all critically ill patients with cancer who underwent brain MRI for acute neurologic symptoms or signs during their ICU stays were ultimately diagnosed with IS. Multiple territorial lesions were commonly observed, but the etiologies of IS were not determined in the majority of patients. In addition, newly diagnosed brain metastases were relatively rare in patients with structural brain lesions. Finally, an abnormal neurological finding (hemiparesis) was found to be independently associated with IS, whereas seizure was inversely associated with IS.

In patients with cancer, IS is often caused by coagulation disorders that are related to the cancer itself or to the cancer treatment. Specifically, coagulation disorders can arise from metastatic lesions to the central nervous system or from vascular injury due to cancer therapy [12–15]. However, several studies have reported that the stroke patterns and vascular risk factors in patients with cancer are not significantly different from those of the general population [23–25]. Therefore, it remains unclear whether the characteristics of IS in patients with cancer differ from those of the general population. However, recent large scale studies of patients with stroke and cancer have concluded that IS in cancer patients is distinct in terms of risk factors, stroke mechanisms, and lesion patterns from IS in the general population [9,14]. In this study, the etiologies in the majority of the critically ill patients with cancer did not correspond to known stroke mechanisms, a finding that is consistent with previous studies. In cancer patients without determined etiologies, cancer-specific mechanisms are likely to be the main cause of IS [13]. Therefore, most strokes in our patients are presumably cancer-related.

The risk factors for IS in critically ill patients with cancer have been proposed to be different from the general risk factors for IS in non-critically ill patients with cancer [26]. Coagulation disorders are common complications in critically ill patients [7]; these disorders may favour thrombosis and intracranial arterial occlusion. In the present study, thrombotic events were more common in patients with IS than in patients without IS, although no significant difference was observed between the two groups regarding hypercoagulability. In addition, multiple disseminated ischemic lesions were commonly observed in the present study.

Acute changes of mental status are common in patients with cancer [27,28]. Potential causes of acute altered mental status in these patients include toxicity, metabolic disorders, drugs, and brain lesions [27]. Structural brain lesions are typically brain metastases in patients with cancer who experience acute changes in mental status [28]. In the present study, approximately two thirds of patients who underwent brain MRI showed acute changes in mental status and half of the patients were ultimately diagnosed with IS. These encephalopathy-mimicking symptoms might be associated with multiple incidences of territorial IS in critically ill patients with cancer. Therefore, brain MRI may be necessary to confirm the suspicion of multiple territorial strokes when critically ill patients with cancer exhibit altered mental status, since many factors can potentially cause mental changes in patients admitted to medical ICUs. However, the symptoms of IS in critically ill patients with cancer may be masked by sedatives or analgesic medications, which are often administered in the ICU. In addition, neuroimaging is not feasible for all critically ill patients with cancer suspected to have IS due to limitations in transport for examination, especially for patients who are mechanically ventilated [29]. Therefore, accurate clinical information on IS in critically ill patients with cancer, especially the likelihood of IS, can help intensivists decide if neuroimaging should be performed. In the present study, hemiparesis was more common in patients with IS than in patients without IS and was also independently associated with IS in a multivariate analysis. Therefore, careful examination and imaging studies may be required when hemiparesis symptoms are observed in critically ill patients with cancer.

Our study does have several potential limitations that should be acknowledged. First, given the observational nature of this study, selection bias may have influenced our findings. Furthermore, our study was conducted at a single institution with a specialized ICU for critically ill patients with cancer. Thus, our findings may not be broadly applicable to other centres at which no experienced intensivists are available for oncological critical care. Second, more than sixty percent of all patients were mechanically ventilated in our study. Sedatives and analgesics were commonly used by these patients, which complicated neurologic examinations. Aphasia and dysarthria are two important neurological signs that may be associated with structural brain lesions. However, it was difficult to confirm these signs in most of the patients who were mechanically ventilated. Third, the presence of carotid artery stenosis could not be evaluated in 26 (30%) patients including 9 patients finally diagnosed as having IS. However, there was no significant stenosis in patients underwent cervical vessels evaluation with MR angiography. Finally, we did not systematically screen all patients with acute neurologic symptoms and/or signs of IS during their ICU stays. Patients who were more severely ill might not have undergone brain MRI, even if they were strongly suspected to have IS. The number of patients with suspected IS who refused further evaluation could not be extracted from the medical records of the study period.

## Conclusions

In conclusion, IS developed during ICU stays in critically ill patients with cancer have particular features that may be associated with cancer-related mechanism. In addition, the results of this study suggest that brain MRI should be performed even in critically ill patients with cancer, especially with risk factors and acute neurologic changes. However, future studies with larger cohorts will be important to confirm this finding.

## References

[1] Barlas I, Oropello JM, Benjamin E. Neurologic complications in intensive care. Curr Opin Crit Care. 2001;7: 68–73. 11373513 10.1097/00075198-200104000-00003

[2] Naik-Tolani S, Oropello JM, Benjamin E. Neurologic complications in the intensive care unit. Clin Chest Med. 1999;20: 423–434, ix 10386265 10.1016/s0272-5231(05)70150-7

[3] Bleck TP, Smith MC, Pierre-Louis SJ, Jares JJ, Murray J, Hansen CA. Neurologic complications of critical medical illnesses. Crit Care Med. 1993;21: 98–103. 8420739 10.1097/00003246-199301000-00019

[4] Isensee LM, Weiner LJ, Hart RG. Neurologic disorders in a medical intensive care unit: A prospective survey. J Crit Care.4: 208–210.

[5] Wilcox ME, Brummel NE, Archer K, Ely EW, Jackson JC, Hopkins RO. Cognitive dysfunction in ICU patients: risk factors, predictors, and rehabilitation interventions. Crit Care Med. 2013;41: S81–98. 10.1097/CCM.0b013e3182a16946 23989098

[6] Osias J, Manno E. Neuromuscular complications of critical illness. Crit Care Clin. 2014;30: 785–794. 10.1016/j.ccc.2014.06.008 25257741

[7] Rogers LR. Cerebrovascular complications in patients with cancer. Semin Neurol. 2010;30: 311–319. 10.1055/s-0030-1255224 20577937

[8] Graus F, Rogers LR, Posner JB. Cerebrovascular complications in patients with cancer. Medicine (Baltimore). 1985;64: 16–35. 3965856 10.1097/00005792-198501000-00002

[9] Kim SG, Hong JM, Kim HY, Lee J, Chung PW, Park KY, et al Ischemic stroke in cancer patients with and without conventional mechanisms: a multicenter study in Korea. Stroke. 2010;41: 798–801. 10.1161/STROKEAHA.109.571356 20150545

[10] Lefkovitz NW, Roessmann U, Kori SH. Major cerebral infarction from tumor embolus. Stroke. 1986;17: 555–557. 3012831 10.1161/01.str.17.3.555

[11] O'Neill BP, Dinapoli RP, Okazaki H. Cerebral infarction as a result of tumor emboli. Cancer. 1987;60: 90–95. 3581035 10.1002/1097-0142(19870701)60:1<90::aid-cncr2820600116>3.0.co;2-c

[12] Cestari DM, Weine DM, Panageas KS, Segal AZ, DeAngelis LM. Stroke in patients with cancer: incidence and etiology. Neurology. 2004;62: 2025–2030. 15184609 10.1212/01.wnl.0000129912.56486.2b

[13] Bang OY, Seok JM, Kim SG, Hong JM, Kim HY, Lee J, et al Ischemic stroke and cancer: stroke severely impacts cancer patients, while cancer increases the number of strokes. J Clin Neurol. 2011;7: 53–59. 10.3988/jcn.2011.7.2.53 21779292 PMC3131539

[14] Lee EJ, Nah HW, Kwon JY, Kang DW, Kwon SU, Kim JS. Ischemic stroke in patients with cancer: is it different from usual strokes? Int J Stroke. 2014;9: 406–412. 10.1111/ijs.12124 23981525

[15] Zhang YY, Chan DK, Cordato D, Shen Q, Sheng AZ. Stroke risk factor, pattern and outcome in patients with cancer. Acta Neurol Scand. 2006;114: 378–383. 17083337 10.1111/j.1600-0404.2006.00709.x

[16] Chong J, Lu D, Aragao F, Singer MB, Schonewille WJ, Silvers A, et al Diffusion-weighted MR of acute cerebral infarction: comparison of data processing methods. AJNR Am J Neuroradiol. 1998;19: 1733–1739. 9802498 PMC8337469

[17] Adams HP Jr., Bendixen BH, Kappelle LJ, Biller J, Love BB, Gordon DL, et al Classification of subtype of acute ischemic stroke. Definitions for use in a multicenter clinical trial. TOAST. Trial of Org 10172 in Acute Stroke Treatment. Stroke. 1993;24: 35–41. 7678184 10.1161/01.str.24.1.35

[18] Darmon M, Thiery G, Ciroldi M, de Miranda S, Galicier L, Raffoux E, et al Intensive care in patients with newly diagnosed malignancies and a need for cancer chemotherapy. Crit Care Med. 2005;33: 2488–2493. 16276171 10.1097/01.ccm.0000181728.13354.0a

[19] Benoit DD, Depuydt PO, Vandewoude KH, Offner FC, Boterberg T, De Cock CA, et al Outcome in severely ill patients with hematological malignancies who received intravenous chemotherapy in the intensive care unit. Intensive Care Med. 2006;32: 93–99. 16308681 10.1007/s00134-005-2836-5

[20] Song JU, Suh GY, Park HY, Lim SY, Han SG, Kang YR, et al Early intervention on the outcomes in critically ill cancer patients admitted to intensive care units. Intensive Care Med. 2012;38: 1505–1513. 10.1007/s00134-012-2594-0 22592633

[21] Yoo H, Suh GY, Jeong BH, Lim SY, Chung MP, Kwon OJ, et al Etiologies, diagnostic strategies, and outcomes of diffuse pulmonary infiltrates causing acute respiratory failure in cancer patients: a retrospective observational study. Crit Care. 2013;17: R150 10.1186/cc12829 23880212 PMC4055964

[22] Taylor FB Jr., Toh CH, Hoots WK, Wada H, Levi M. Towards definition, clinical and laboratory criteria, and a scoring system for disseminated intravascular coagulation. Thromb Haemost. 2001;86: 1327–1330. 11816725

[23] Zhang YY, Cordato D, Shen Q, Sheng AZ, Hung WT, Chan DK. Risk factor, pattern, etiology and outcome in ischemic stroke patients with cancer: a nested case-control study. Cerebrovasc Dis. 2007;23: 181–187. 17143001 10.1159/000097639

[24] Stefan O, Vera N, Otto B, Heinz L, Wolfgang G. Stroke in cancer patients: a risk factor analysis. J Neurooncol. 2009;94: 221–226. 10.1007/s11060-009-9818-3 19280119

[25] Chaturvedi S, Ansell J, Recht L. Should cerebral ischemic events in cancer patients be considered a manifestation of hypercoagulability? Stroke. 1994;25: 1215–1218. 8202983 10.1161/01.str.25.6.1215

[26] Pilato F, Profice P, Dileone M, Ranieri F, Capone F, Minicuci G, et al Stroke in critically ill patients. Minerva Anestesiol. 2009;75: 245–250. 18636061

[27] Tuma R, DeAngelis LM. Altered mental status in patients with cancer. Arch Neurol. 2000;57: 1727–1731. 11115238 10.1001/archneur.57.12.1727

[28] Doriath V, Paesmans M, Catteau G, Hildebrand J. Acute confusion in patients with systemic cancer. J Neurooncol. 2007;83: 285–289. 17225935 10.1007/s11060-006-9319-6

[29] Schwebel C, Clec'h C, Magne S, Minet C, Garrouste-Orgeas M, Bonadona A, et al Safety of intrahospital transport in ventilated critically ill patients: a multicenter cohort study*. Crit Care Med. 2013;41: 1919–1928. 10.1097/CCM.0b013e31828a3bbd 23863225

